# Replacing cottonseed meal and sorghum with dried distillers’ grains with solubles enhances the growth performance, carcass traits, and meat quality of feedlot lambs

**DOI:** 10.1093/tas/txac040

**Published:** 2022-04-13

**Authors:** Danilo G Quadros, Chris R Kerth

**Affiliations:** Texas A&M AgriLife Research, 7887 U.S. Hwy 87 N, San Angelo, TX 76901, USA; Department of Animal Science, Texas A&M University, College Station, TX 77843, USA

**Keywords:** carcass, fatty acids, intake, Rambouillet, sensory panel, wool

## Abstract

We evaluated the impacts of substituting cottonseed meal (CSM) and sorghum grain (SG) with dried distillers` grains with solubles (DDGS) in lamb feedlot diets on the dry matter intake (DMI), the growth performance, blood serum analysis, feces phosphorus (P) and nitrogen (N), wool production and quality, carcass traits, adipose tissue fatty acid (FA) profiles, and sensory panel tests. For 72 d, Rambouillet wether lambs (*n* = 44, initial body weight, BW = 28.8 ± 3.3 kg) were individually fed ad libitum pelleted diets containing DDGS that replaced 0% (0DDGS), 25% (25DDGS), 50% (50DDGS), or 75% (75DDGS) of the CSM and SG in a completely randomized design trial. Linear and quadratic effects of DDGS levels on the response variables were analyzed. Treatment × day interactions (*P <* 0.001) were observed for BW and DMI. As the DDGS level was increased, DMI (from days 21 to 70), lamb BW (from days 56 to 70), average daily gain, blood urea nitrogen and P, and fecal P linearly increased (*P* ≤ 0.05). Fecal N quadratically increased (*P =* 0.01), but no effects were found for gain:feed, blood insulin-like growth factor-1, or calcium. No differences in wool production or most of the wool quality parameters were detected. Adipose tissue stearic acid linearly increased (*P =* 0.02), linoleic acid quadratically increased (*P =* 0.01), and oleic acid tended to quadratically decrease (*P* = 0.08) as the DDGS increased in the diets. Increasing the DDGS level in the diets quadratically increased the hot carcass weight (*P* = 0.02), backfat thickness (*P* = 0.04), and body wall thickness (*P* < 0.001) while having no impact on the longissimus muscle area. As the DDGS increased in the diet, juiciness, tenderness, and overall acceptability linearly increased (*P* ≤ 0.05), while having no effect on the cook-loss, flavor intensity, or off-flavor detectability. Replacing 50% of CSM and SG with DDGS improved growth performance and enhanced the carcass and meat quality.

## Teaser

This research will guide animal nutritionists to maximize the inclusion of DDGS in feedlot lamb diets to increase growth performance and enhance carcass traits and meat quality.

## INTRODUCTION

The corn ethanol industry has promoted the ready availability of distillers` grains co-product. In the last 15 years, the dried distillers’ grains with solubles (DDGS) supply in the U.S. has increased approximately 3.5-fold, reaching 35 million metric tons in 2020/2021 (USDA, 2021). Due to its crude protein (CP; 26%–33%; Liu, 2011) and energy contents (89.7% of total digestible nutrients, TDN; Nuez Ortín and Yu, 2009) as well as its lower relative costs (Alshdaifat and Obeidat, 2019), DDGS has been included in animal diets to replace traditional feedstuffs such as soybean meal (SBM) and corn (Klopfenstein, 1996; Hoffman and Baker, 2011; Karaca et al., 2021). Cottonseed meal (CSM) and sorghum grain (SG) are common feed ingredients used in livestock diets that can also be replaced by DDGS in small ruminant production systems, notably for fattening lambs (Whitney and Braden, 2010; Hodges et al., 2020a). Furthermore, DDGS contains high levels of rumen undegradable protein (RUP; 45% of total protein; Belyea et al., 2010) and sulfur (S, 0.11%-0.84%; Liu, 2011), which can enhance growth and wool production (Hassan et al., 1991; Castro-Pérez et al., 2014), and fiber that help reduce acidosis problems (Klopfenstein, 1996). Maximizing the inclusion rate of DDGS in small ruminant diets without negatively affecting growth, health, or end products will benefit not only corn growers and the ethanol industry but also the sheep industry by potentially reducing feed costs (Felix et al., 2012; Hodges et al., 2020b; Quadros et al., 2021).

It is hypothesized that DDGS can partially replace CSM and SG in feedlot lamb diets without compromising production, physiological parameters, or meat quality. Therefore, this study aimed to evaluate the effects of replacing CSM and SG with DDGS in Rambouillet wether diets on lamb growth factors, wool quality and production, and as meat quality.

## MATERIALS AND METHODS

### Animals and Management

The experimental protocol was approved by the Texas A&M University Institutional Animal Care and Use Committee (2018-013A).

Rambouillet wether lambs (*n* = 44; approximate age = 4 mo; initial body weight, BW = 28.9 ± 3.3 kg) were weighed, stratified by BW, and randomly assigned to one of four diets (*n* = 11/treatment). The trial was conducted following a completely randomized design using the animal as experimental unit. Lambs received an ear tag and a subcutaneous injection of a clostridial vaccine (Vision7 with SPUR, Inervet Inc., Millsboro, DE) and were randomly assigned to an individual, completely covered dirt pen (2.44 × 2.97 m) with feed bunks and automatic watering systems. The adaptation period to the experimental diets was conducted in two steps and consisted of increasing levels of concentrate (50% and 65%, dry matter, DM, basis) and DDGS in the diet over 16 days (8 days for each step, using 50% and 100% of the proportion of DDGS planned for each diet, respectively) until reaching the levels of concentrate (20:80) in the finishing experimental diets according to the assigned treatment group. Simultaneously, the non-pelleted feed was gradually replaced by pelleted treatment diets. Pelleted diets contained corn DDGS that replaced 0% (0DDGS), 25% (25DDGS), 50% (50DDGS), or 75% (75DDGS, DM basis) of the ground SG and CSM (Table 1). Monensin (22 g/metric ton of Rumensin 80; Elanco, Indianapolis, IN) was included in all diets. Lambs were individually fed once daily at 0800 hours, with an allowance of approximately 10% refusal. Feed refusals were collected three times per week and weighed to determine the average daily DM intake (DMI). Lamb BW was recorded on day 0 and then every 7 days until day 70. The animals were weighed at the same time each week. The average daily gain (ADG) and average daily DMI were determined between days in which BW was recorded; the gain:feed ratio (G:F) was calculated by dividing ADG by DMI.

**Table 1.** Ingredient and chemical composition (% DM basis) of dried distillers` grains with solubles (DDGS) and treatment diets

Item, %^1^	DDGS	Diet^2^			
		0DDGS	25DDGS	50DDGS	75DDGS
Cottonseed hulls		20.00	20.00	20.00	20.00
DDGS		0.00	17.58	35.16	52.74
Cottonseed meal		10.00	7.50	5.00	2.50
Ground sorghum grain		60.72	45.44	30.17	14.86
Cane molasses		4.00	4.00	4.00	4.00
Limestone		1.52	2.18	2.82	3.50
Ammonium chloride		0.95	0.95	0.95	0.95
Salt		0.95	0.95	0.95	0.95
Urea		1.36	0.90	0.45	0.00
Mineral and vitamin premix		0.50	0.50	0.50	0.50
Chemical composition					
CP	25.1	17.6	18.7	21.5	21.1
Crude fat	5.0	3.5	5.2	7.1	6.6
NDF	37.1	18.9	25.6	28.3	37.1
ADF	13.9	12.2	16.6	18.7	23.8
Ash	6.2	4.9	4.8	5.4	7.2
NEm, Mcal/kg	2.05	1.66	1.66	1.65	1.64
NEl, Mcal/kg	1.28	1.07	1.05	1.03	1.00
Ca	0.1	1.7	1.3	1.5	1.9
P	1.1	0.3	0.3	0.6	0.7
Ca:P ratio	0.09	5.6	4.1	2.7	2.6
Mg	0.45	0.19	0.29	0.30	0.34
K	1.92	0.79	1.42	1.07	1.29
Na	0.68	0.14	0.26	0.22	0.35
S	0.44	0.19	0.31	0.36	0.40

Mineral and vitamin premix = sodium chloride, potassium chloride, sulfur, manganous oxide, zinc oxide, vitamins A, D, and E, calcium carbonate, cottonseed meal, cane molasses, and animal fat. CP = crude protein; NDF = neutral detergent fiber; ADF = acid detergent fiber.

Pelleted diets contained DDGS that replaced 0% (0DDGS), 25% (25DDGS), 50% (50DDGS), or 75% (75DDGS) of the cottonseed meal and sorghum grain.

### Feed and Feces Collection and Analysis

Samples of the feed ingredients in each treatment diet were randomly collected, dried at 55 °C in a forced-air oven for 48 h, ground in a Wiley Mill (Arthur H. Thomas Co., Philadelphia, PA) to pass a 1-mm screen, and stored at −20 °C until analysis. Nitrogen (N) content was analyzed by a standard method (990.03, AOAC 2006), and the CP was calculated as 6.25 × N. The crude fat (CF) was analyzed by ether extraction (2003.05, AOAC 2006), and the ash analysis was determined by a standard method (942.05, AOAC 2006). The neutral detergent fiber (NDF) and acid detergent fiber (ADF) were analyzed using the Van Soest et al. (1991) procedure modified for an Ankom 2000 Fiber Analyzer (Ankom Technol. Corp., Fairport, NY) without correcting for residual ash. The feed S was evaluated by a Leco (model SC-432, St. Joseph, MI) analyzer, and all other minerals were analyzed by a Thermo Jarrell Ash IRIS Advantage HX Inductively Coupled Plasma Radial Spectrometer (Thermo Instrument Systems, Inc., Waltham, MA).

Feces were rectally collected 5 h after feeding on day 60 and analyzed for phosphorus (P) content using a Thermo iCAP 6300 inductively coupled plasma radial spectrometer (Thermo Instrument Systems) according to the procedure outlined in Wolf et al. (2003), and the N content was analyzed by a Leco analyzer (model CN-628; Leco Corporation, St. Joseph, MI).

### Blood Collection and Analysis

A 10-mL blood sample was collected from each lamb on days 0, 14, 28, 42, and 70, 4 h after feeding via jugular venipuncture using a non-heparinized vacutainer collection tube (serum separator tube, gel and clot activator; Becton Dickenson, Franklin Lakes, NJ). The blood samples were allowed to clot for approximately 30 min at room temperature and then centrifuged (Beckman Coulter TJ6 refrigerated centrifuge, Fullerton, CA) at 970 × *g* for 25 min at 4 °C. Serum was decanted and frozen at −20 °C until analysis. The blood urea N (BUN) was analyzed using a commercial kit (Teco Diagnostics, Anaheim, CA), and blood insulin-like growth factor-1 (IGF-1) was determined by radioimmunoassay using the procedure outlined by Berrie et al. (1995). The serum calcium (Ca) and P contents were analyzed from days 0, 14, 42, and 70 samples using an Olympus AU400E analyzer (Olympus America Inc., Center Valley, PA).

### Wool Production and Quality

Lambs were shorn 2 days before study initiation and on day 65. Fleece and fiber measurements were made in the Wool and Mohair Laboratory at the Texas AgriLife Research Center, San Angelo. After grease fleece weights were obtained for each fleece, staples (*n* = 10) were removed from random positions in each fleece for staple strength (Agritest Pty Ltd., Sidney, NSW) and length measurements (D1234; ASTM, 2007). The remainder of the fleece was pressure-cored (32 × 13 mm cores, Johnson and Larsen, 1978) to obtain a 50-g random sample. Two 25-g sub-samples were used to determine the laboratory scoured yield (D584; ASTM, 2007). One of the washed and dried duplicates was mini-cored (D6500; ASTM, 2008) to obtain a few milligrams of 2-mm snippets that represented the whole fleece. Snippets were washed in a Buchner funnel with 1,1,1-trichloroethane (10 mL) and acetone (10 mL), dried at 105 °C for 1 h, cooled, and conditioned for 12 h at the standard atmosphere of 21 ± 1 °C and 65 ± 2% relative humidity (D1776; ASTM, 2007). Conditioned snippets were then spread onto microscope slides (7 × 7 cm) and measured for fiber diameter distribution (mean, SD, and CV), comfort factor (% fibers ≤ 30 µm), and average fiber curvature (mean, SD, and CV) using an OFDA 100 (BSC Electronics, Ardross, Western Australia; Baxter et al., 1992; ASTM, 2008). Wool production per unit of BW (g/kg) was calculated as clean wool production divided by final shorn BW.

### Carcass Characteristics and the Feed and Meat Fatty Acid Profile

On day 72, lambs were transported 500 m to the Angelo State University Food Safety and Product Development Laboratory and humanely harvested after a 24-h fast. The shrunk BW and hot carcass weight (HCW) were recorded, and the carcasses were chilled at 2 ± 1 °C. At 48 h postmortem, each carcass was ribbed between the 12th and 13th ribs and analyzed to determine the longissimus muscle area (LMA), the backfat thickness (BFT) at the 12th rib, the body wall thickness (BWT), and the leg circumference (LC) across the stifle joint (USDA, 1997). The longissimus muscle (LM) was removed from the left side of each carcass by deboning from the thoracic vertebrae according to procedures of the North American Meat Processors (#232a; NAMP, 1997). Five 2.54-cm-thick chops were cut starting from the posterior end. The first chop was designated for fatty acid (FA) methyl ester (FAME) analysis, cut to straighten the LM face, vacuum-packaged separately, and stored at −80 °C. Subsequently, four 2.54-cm-thick chops were serially cut for sensory analysis, labeled, vacuum packaged, and stored at −10 °C.

The subsample collected from the LM cross-section, including any residual intermuscular fat, and feed samples were pulverized in liquid nitrogen. Then, total lipids were extracted by a modification of the method outlined in Folch et al. (1957). Adipose tissue (100 mg) was extracted in chloroform:methanol (2:1, vol/vol), and FAME analysis was prepared as described by Morrison and Smith (1964) but modified to include an additional saponification step (Archibeque et al., 2005). The FAME analysis was conducted using a Varian gas chromatograph (GC; model CP-3800 fixed with a CP-8200 autosampler; Varian, Inc., Walnut Creek, CA). Separation of the FAME was accomplished on a fused silica capillary column CP-Sil88 (100 m long × 0.25 mm i.d.; Chrompack, Inc., Middleburg, the Netherlands; helium as carrier gas and flow rate = 1.2 mL/min). After 32 min at 180 °C, the oven temperature was increased at 20 °C/min to 225 °C and held for 13.75 min; the total run time was 48 min. The injector and detector temperatures were 270 and 300 °C, respectively. Individual FA was identified using genuine external standards (Nu-Chek Prep, Inc., Elysian, MN).

### The Sensory Panel Evaluation

A trained sensory panel (6 members; Cross et al., 1978) evaluated the chops cut from the loin section (AMSA, 1995). Randomly selected chops were thawed for 24 h at 2 ± 1 °C and cooked on a clam-shell-style grill (Kerth et al., 2003) for 7 min. Samples were trimmed to less than 0.64 cm of outside fat and connective tissue, cut into 1.27-cm × 1.27-cm portions and placed in warming pans until served to the panelists. The cook-loss was expressed as a percentage of post-cooking weight loss from the raw weight. Samples from each chop were evaluated by panelists who were secluded in partitioned booths with a controlled level of red incandescent light. A “warm-up” sample chop was served at the initiation of each sensory session, followed by 6 × 8 chop samples per session. Panelists were instructed to cleanse their palates with a salt-free saltine cracker and water before each sample. Chops were evaluated for initial and sustained juiciness, initial and sustained tenderness, and flavor intensity on a scale of 1 to 8, where 1 = extremely dry, tough, and bland, and 8 = extremely juicy, tender, and intense, respectively. Chops were also evaluated for off-flavor (1 = extreme off-flavor and 4 = no off-flavor) and overall acceptability (1 = not acceptable and 8 = highly acceptable).

### Statistical Analysis

Lamb BW, ADG, DMI, G:F, and blood serum variables were analyzed by ANOVA using the PROC MIXED procedure of SAS (SAS Inst. Inc., Cary, NC) with a model that included treatment, day, and treatment × day interaction; the day was a repeated measure, and individual lambs were the subject. When a treatment × day interaction was observed (*P* ≤ 0.05), effects were evaluated by day. Suitable covariance structures were compared for each model. Fecal, wool, carcass, sensory, and meat FA profiles were analyzed using a model that included the lamb as the experimental unit. The average fiber diameter of the mid-side sample at the start of the study was used as the covariate for the average fiber diameter of the fleece, and the initial BW was used as a covariate for the clean fleece weight, but these covariates were later removed because they were not significant. The percentage of fat in the meat samples was analyzed by ANOVA using the GLIMMIX procedure of SAS (β distribution) with a model that included the diet as the fixed effect and the lamb as the experimental unit. All data are reported as least squares means with the greatest SEM. Treatment effects were tested using the following single degree of freedom orthogonal polynomial contrasts: linear and quadratic effects of replacing CSM and SG with DDGS. Only the highest-order contrast that was significant (*P* < 0.10) will be discussed further in this paper. Statistical significance was declared at *P* ≤ 0.05 and a tendency at 0.05 < *P* ≤ 0.10.

## RESULTS AND DISCUSSION

### The Chemical Composition and FA Profiles of the Treatment Diets

The chemical composition of the DDGS and diets is presented in Table 1, and the FA profiles of DDGS, CSM, SG, and diets are presented in Table 2. The DDGS is an advantageous feed because it can substitute traditional protein (i.e., SBM, CSM; Hoffman and Baker, 2011, Castro-Pérez et al., 2014; Karaca et al., 2021) and high-energy containing (i.e., corn, SG; Klopfenstein, 1996; Hodges et al., 2020a) feedstuffs in livestock diets; however, its composition can vary considerably depending on the processing method (Böttger and Südekum, 2018). The CP variability in the diets was due to a greater CP content in the DDGS compared with SG (Trujillo et al., 2016), as the CSM CP levels were low (Table 1). Urea added in 0DDGS and 25DDGS did not turn diets isoproteic and adding more urea could bring metabolic risks and alter the experimental results.

**Table 2.** Fatty acid (FA) profile of sorghum grain (SG), cottonseed meal (CSM), dried distillers` grains with solubles (DDGS), and treatment diets

Item^1^	SG	CSM	DDGS	Diet^2^			
				0DDGS	25DDGS	50DDGS	75DDGS
Fat, %	4.8	6.8	5.3	3.8	4.3	5.0	5.3
FA, %							
Lauric acid (C12:0)	8.1	8.6	10.6	11.1	9.5	8.3	9.4
Palmitic acid (C16:0)	12.5	20.1	14.9	13.9	14.1	13.9	14.6
Palmitoleic acid (C16:1)	0.40	0.40	nd	0.39	0.37	0.34	0.30
Stearic acid (C18:0)	1.7	2.9	2.7	1.7	1.9	1.9	2.1
Oleic acid (C18:1)	1.2	0.7	0.9	1.1	1.1	1.1	1.0
Oleic acid (C18:1c9)	32.3	18.6	19.1	24.8	24.7	25.1	23.0
Linoleic acid (C18:2)	41.9	47.2	48.5	45.5	46.2	46.9	47.0
Linolenic acid (C18:3)	1.9	0.8	2.5	1.8	2.0	2.1	2.1
Arachidonic acid (C20:4)	Nd	0.30	0.26	nd	nd	0.21	0.25
Lignoceric acid (C24:0)	Nd	nd	0.35	nd	0.24	0.24	0.26

FA = expressed as a percentage of total FA. nd = none detected.

Pelleted diets contained DDGS that replaced 0% (0DDGS), 25% (25DDGS), 50% (50DDGS), or 75% (75DDGS) of the cottonseed meal and sorghum grain.

Increasing the DDGS levels in the diets increased NDF and ADF (Table 1) due to greater NDF and ADF contents in the DDGS compared to the SG (Trujillo et al., 2016). The CF in the DDGS was less than 8.2%-11.4% range obtained by Spiehs et al. (2002) but slightly greater than 4.4% observed by Whitney and Lupton (2010). The CF increased with the DDGS level in the diets, but the FA profile of the diets did not follow the same trend, with discrete changes in the major FA, such as C18:2, C18:1c9, C12:0, and C12:0 (Table 2). According to Moreau et al. (2011), the major FA in DDGS is linoleic acid (C18:2), followed by oleic acid (C18:1) and palmitic acid (C16:0), while stearic (C18:0) and linolenic (C18:3) acids are present at low levels.

### Growth Performance

Treatment × day interactions were observed (*P* ≤ 0.05) for BW and DMI (Table 3). After 21 days, increasing DDGS levels in the diets linearly increased DMI (*P* ≤ 0.02), similar to what was observed by Schauer et al. (2008) when replacing approximately 70% of barley and all SBM with DDGS in Rambouillet wether and ewe lamb diets. Conversely, McEachern et al. (2009) observed no effect of substituting all CSM with DDGS on the DMI of Rambouillet wether lambs. Increased the NDF in experimental diets (Table 1) did not constrain the DMI (Table 3), probably because the physically effective NDF of DDGS provided little effective fiber (Kleinschmit et al., 2007).

**Table 3.** Effects of substituting dried distillers` grains with solubles (DDGS) for cottonseed meal and sorghum grain on lamb growth performance, blood urea N (BUN), serum Ca, P and insulin-like growth factor-1 (IGF-1), and fecal P and N concentrations

Item^1^	Diet^2^				SEM^3^	*P*-value^4^	
	0DDGS	25DDGS	50DDGS	75DDGS		L	Q
BW, kg							
Day 0	28.9	28.3	29.0	29.3	1.1	0.66	0.67
Day 7	30.9	31.4	31.6	30.7	1.1	0.95	0.50
Day 14	33.2	33.3	33.8	32.8	1.1	0.91	0.60
Day 21	34.3	34.8	35.5	34.8	1.1	0.62	0.58
Day 28	35.1	36.2	37.5	36.4	1.2	0.32	0.35
Day 35	37.1	37.7	39.4	38.4	1.1	0.21	0.48
Day 42	38.5	38.9	40.8	40.0	1.2	0.19	0.58
Day 49	40.1	40.4	43.1	41.5	1.2	0.16	0.41
Day 56	41.1	42.6	44.5	44.5	1.3	0.03	0.54
Day 63	42.3	43.8	46.3	46.1	1.3	0.02	0.48
Day 70	42.8	44.0	46.7	45.9	1.3	0.05	0.45
ADG, kg	0.20	0.21	0.26	0.24	0.01	0.001	0.24
DMI, kg							
Day 7	1.28	1.29	1.36	1.17	0.08	0.43	0.22
Day 14	1.37	1.44	1.61	1.41	0.08	0.43	0.11
Day 21	1.23	1.43	1.70	1.65	0.08	<0.001	0.10
Day 28	1.26	1.37	1.57	1.65	0.09	0.002	0.84
Day 35	1.25	1.34	1.70	1.64	0.07	<0.001	0.22
Day 42	1.33	1.35	1.64	1.76	0.07	<0.001	0.49
Day 49	1.27	1.35	1.66	1.74	0.08	<0.001	0.99
Day 56	1.27	1.43	1.68	1.70	0.09	<0.001	0.42
Day 63	1.11	1.24	1.46	1.58	0.09	<0.001	0.99
Day 70	1.34	1.45	1.65	1.58	0.09	0.02	0.31
G:F, kg/kg	0.16	0.16	0.17	0.15	0.01	0.68	0.27
BUN, mg/dL	13.1	14.6	18.4	17.8	0.8	<0.001	0.17
IGF-1, ng/mL	217.7	221.0	250.4	227.8	13.4	0.30	0.33
Ca, mg/dL	10.1	9.8	9.9	9.4	0.3	0.13	0.64
P, mg/dL	7.7	9.1	9.3	9.9	0.4	<0.001	0.25
Fecal P, %	0.39	0.70	0.78	1.23	0.10	<0.001	0.48
Fecal N, %	2.9	3.6	3.6	3.6	0.1	<0.001	0.01

Treatment × day interactions: BW (*P* < 0.001); ADG (*P* = 0.08); DMI (*P* < 0.001); G:F (*P* = 0.68); BUN (*P* = 0.43); IGF-1 (*P* = 0.20); serum Ca (*P* = 0.93); serum P (*P* = 0.60).

Lambs were fed pelleted diets containing DDGS that replaced 0% (0DDGS), 25% (25DDGS), 50% (50DDGS), or 75% (75DDGS) of the cottonseed meal and sorghum grain.

SEM represents the greatest standard error of the mean.

Linear (L) and quadratic (Q) orthogonal polynomial contrasts.

The BW from days 0 to 49 was similar (*P* > 0.16) among treatments regardless of DDGS level. However, from days 56 to 70, increasing DDGS in the diets promoted a linear increase (*P* ≤ 0.05) in BW. Assuming a 51% RUP in DDGS and CSM (McEachern et al., 2009), 75DDGS diets contained 2.9 times more RUP than 0DDGS diets, which probably improved protein utilization and animal productivity (Belyea et al., 2010). In addition, the additional CF with the increasing DDGS levels likely increased the net energy for gain (Schauer et al., 2008).

The ADG increased linearly as the DDGS level in the diets increased (*P* < 0.001; Table 3), corroborating Castro-Pérez et al. (2014) who replaced up to 45% of the dry-rolled corn and SBM with DDGS in the diets of Pelibuey × Katahdin crossbred intact male lambs. However, several authors (Huls et al., 2006; Şahin et al., 2013; Crane et al., 2017) found no differences in ADG when substituting traditional protein and energy feedstuffs with DDGS, but it should be noted that the DDGS proportions in the diets tested by those authors were up to 30%, while in this trial surpassed 50%. When higher levels (~60%) of DDGS were tested (Felix et al., 2012; Curzaynz-Leyva et al., 2019; Hodges et al., 2020a), the quadratic effect is more consistent, indicating that partial replacement of traditional protein and energy feedstuffs with DDGS can maximize growth performance.

The G:F was not affected by the diet × day interaction (*P* > 0.05) or diet (*P* > 0.27), with an average of 0.16 ± 0.01 (Table 3), in agreement with the studies by Schauer (2008), McEachern et al. (2009), and Castro-Pérez et al. (2014). Therefore, taking into consideration that DDGS may be potentially cheaper than traditional feedstuffs such as CSM and SG (Obeidat, 2018; Quadros et al., 2021), the absence of a difference in G:F *per se* can increase profitability because the net margin of a feedlot is generally low and the most representative operational cost comes from feed acquisition (Lima et al., 2017).

### Blood and Fecal Analysis

The BUN, serum IGF-1, Ca and P, and fecal P were not influenced by the diet × day interaction (*P* > 0.05). The linear increase in BUN with DDGS levels (*P* < 0.001; Table 3) may be attributed to an increase in rumen degradable protein (RDP) intake (McEachern et al., 2009; Swanson et al., 2000; Hodges et al., 2020a). Additionally, with the increased DMI, the estimates of RDP also increased 80 g/kg from the control group (0DDGS) to the 75DDGS treatment group. Increasing the solubility or degradability of dietary protein can lead to increased ruminal ammonia concentrations, resulting in increased BUN concentrations (Huntington and Archibeque, 2000; Piccione et al. 2006). The increased rumen ammonia concentration with DDGS inclusion in the diets might be beneficial to the metabolic activity of ruminal proteolytic microflora and hence to the extent of dietary protein hydrolysis (Radev, 2012). In addition, the transport of amino acids (AA) and peptides from the small intestine contribute to the absorption of N into the bloodstream (Kohn et al., 2005). The intestinal digestibility of most AA in DDGS products exceeds 92%, and AA availability can be comparable to that of SBM (Mjoun et al., 2010).

As the DDGS levels in the diets increased, fecal N increased quadratically (*P* = 0.01), probably influenced by a greater DMI and N intake. It has been reported that there is a positive correlation between organic matter intake and fecal N excretion in sheep (Peripolli et al., 2011). Compared to the control group (0DDGS), fecal N increased (*P* < 0.001) by almost 25% in the 75DDGS treatment group, which may be related to greater N losses in the RDP due to not being utilized for microbial growth (Mikolayunas-Sandrock et al., 2009).

According to Whitney and Muir (2010) and Yang et al. (2019), IGF-1 synthesis and secretion, which are stimulated by the growth hormone, have a positive correlation with DMI and growth performance. Because the DDGS level did not impact the serum IGF-1 concentration it can be hypothesized that it did not affect the DMI and the growth performance positive responses either. However, a discrepancy between circulating IGF-1 and ADG was found by Whitney and Lupton (2010). Similar to this work, McEachern et al. (2009) did not find a correlated response between serum IGF-1 and growth. These contradictory data in the literature indicate the necessity of further investigation.

Increasing DDGS levels in the diets linearly increased serum P (*P* < 0.001) and fecal P (*P* < 0.001), while serum Ca was independent of the DDGS level (*P* > 0.13; Table 3). This was related to increased P concentration in the diets (Table 1), agreeing with the findings of Villalba et al. (2008). Unusually, serum P surpassed the Ca concentration in 75DDGS (Stojković et al., 2011; Whitney et al., 2014; Song et al., 2018). Serum Ca values were in the range of 9.3–11.7 mg/dL reported by Jianu et al. (2013) for sheep, although P was greater (4.0–7.3 mg/dL). The P levels in the diets with DDGS (Table 3) ranged between 2- and 3-fold above nutritional requirements (NRC, 2007) which is a concern because it can contribute to environmental pollution generated by animal feeding operations (Huls et al., 2006) and can cause a high incidence of urolithiasis (Riedi et al., 2018). Despite the addition of limestone and ammonium chloride to regulate the Ca:P ratio and prevent urinary calculi (Felix et al., 2012), one lamb fed 50DDGS and two lambs fed 75DDGS died near the end of the trial after exhibiting symptoms related to obstructive urolithiasis. The data from these lambs were kept in the dataset. Increased fecal P (Table 3) is related to P intake and absorption (Louvandini and Vitti, 1996), as ruminants primarily excrete P through feces, which is composed of unabsorbed dietary P and endogenous P (Bravo et al., 2003).

### Wool Characteristics

The grease fleece weight was not affected by experimental diets with an average weight of 0.88 kg (Table 4). The obtained daily grease fleece growth rate of 13.5 g/day was slightly less than 15 g/day obtained by McEachern et al. (2009) who tested replacing CSM with DDGS in Rambouillet feedlot lamb diets; however, when considering the clean wool fiber percentage, the clean fleece daily growth rate was the same (6.3 g/day). Clean wool production per unit of BW tended to (*P* = 0.06) quadratically decrease when DDGS was increased in the diets, reaching the lowest point in the 25DDGS experimental group.

**Table 4.** Effects of substituting dried distillers grains for cottonseed meal and sorghum grain on wool characteristics

Item/day^1^	Diet^2^				SEM^3^	*P*-value^4^	
	0DDGS	25DDGS	50DDGS	75DDGS		L	Q
Grease fleece weight, kg	0.86	0.83	0.93	0.91	0.05	0.24	0.99
Clean wool fiber present, %	50.4	44.8	45.9	45.5	2.1	0.12	0.19
Clean fleece weight, kg	0.43	0.36	0.43	0.42	0.03	0.82	0.32
Clean wool production/BW, g/kg	10.3	8.5	9.4	9.9	0.7	0.88	0.06
Average fiber diameter, µm	18.7	18.8	18.9	18.7	0.4	0.95	0.67
SD fiber diameter, µm	4.1	5.2	4.6	4.0	0.5	0.53	0.04
Average staple length, mm	22.4	20.3	22.3	22.5	1.2	0.62	0.29
SD staple length, mm	3.0	2.9	3.2	3.2	0.4	0.61	0.87
Average fiber curvature, deg/mm	105.8	116.6	107.5	110.8	4.5	0.75	0.38
SD fiber curvature, deg/mm	63.6	69.3	65.2	65.7	1.8	0.74	0.12

Lambs were shorn on day 65.

Lambs were fed pelleted diets containing DDGS that replaced 0% (0DDGS), 25% (25DDGS), 50% (50DDGS), or 75% (75DDGS) of the cottonseed meal and sorghum grain.

SEM represents the greatest standard error of the mean.

Linear (L) and quadratic (Q) orthogonal polynomial contrasts.

The fiber diameter is an important raw wool characteristic because it is a significant determinant of greasy wool price, and there is a premium paid for finer wool types (Nolan et al., 2014). The effects of DDGS inclusion were not detected on fiber diameter but quadratically increased (*P* = 0.04) SD fiber diameter, with the highest SD in the 25DDGS experimental group (Table 4).

All other wool characteristics were not influenced by DDGS levels in the diets. Accordingly, Crane et al. (2017) observed no effects of up to 30% DDGS in Suffolk × Rambouillet lamb diets on the fiber diameter distribution, the fiber curvature distribution, the staple length, or the comfort factor. The fiber diameter and fiber elongation rate (i.e., staple length, Table 4) could have corresponded to a higher nutrition plane (Naderi et al., 2015), indicated by increased DMI and ADG (Table 3), as well as changes in the protein source (Ružić-Muslić et al., 2016), despite conflicting results on the relationship between DMI and wool growth (Khan et al., 2012) and no correlation between lamb growth performance and wool quality traits (Malau-Aduli et al., 2019).

### Carcass Characteristics

Increasing the DDGS levels in the diets quadratically increased shrunk BW (*P* = 0.03) and HCW (*P* = 0.02; Table 5), corroborating with Hodges et al. (2020b) who tested replacing CMS and SG with DDGS in feedlot Dorper intact male lamb diets.

**Table 5.** Effects of substituting dried distillers` grains with solubles (DDGS) for cottonseed meal and sorghum grain on lamb carcass traits and adipose tissue fatty acid composition (%, weight basis)

Item^1^	Diet^2^				SEM^3^	*P*-value^4^	
	0DDGS	25DDGS	50DDGS	75DDGS		L	Q
Carcass traits							
Shrunk BW, kg	37.8	40.2	42.2	39.1	1.2	0.27	0.03
HCW, kg	21.9	23.2	24.5	22.5	0.70	0.34	0.02
LMA, cm^2^	14.4	14.8	15.6	14.4	0.5	0.66	0.14
Backfat, cm	0.47	0.52	0.55	0.34	0.07	0.21	0.04
Body wall, cm	1.44	1.70	1.72	1.38	0.09	0.71	<0.001
LC, cm	30.4	31.0	31.6	30.7	0.4	0.32	0.06
Fatty acids							
Fat, %	4.48	3.44	4.46	4.16	0.37	0.87	0.28
Saturated fatty acids, %							
Palmitic acid (16:0)	19.9	19.5	18.3	20.0	0.74	0.78	0.20
Lauric acid (12:0)	16.0	17.9	15.9	17.0	1.1	0.81	0.70
Stearic acid (18:0)	11.4	12.2	12.6	13.3	0.56	0.02	0.88
Myristic acid (14:0)	1.58	1.44	1.31	1.51	0.13	0.56	0.20
Heptadecanoic acid (17:0)	1.21	1.04	0.86	0.95	0.07	0.002	0.05
Polyunsaturated fatty acids, %							
Linoleic acid (C18:2n-6)	6.90	7.91	10.93	6.82	0.94	0.50	0.01
Arachidonic acid (20:4n-6)	1.75	1.75	2.33	1.31	0.48	0.72	0.29
Monounsaturated fatty acids, %							
Oleic acid (18:1n-9)	31.8	30.5	28.6	31.6	1.21	0.64	0.08
*Cis*-vaccenic acid (18:1n-7)	3.46	2.69	3.45	2.76	0.32	0.34	0.90
Vaccenic acid (18:1trans-11)	1.52	1.43	1.23	1.12	0.07	<0.001	0.89
Palmitoleic acid (16:1n7)	1.21	1.17	0.93	1.10	0.09	0.14	0.22

Shrunk BW = BW without the fleece; HCW = hot carcass weight; REA = ribeye area; LC = leg circumference; Fatty acids were extracted from the Longissimus dorsi.

Lambs were fed 80% concentrate pelleted diets containing DDGS that replaced 0% (0DDGS), 25% (25DDGS), 50% (50DDGS), or 75% (75DDGS) of the cottonseed meal and sorghum grain.

SEM represents the greatest standard error of the mean.

Linear (L) and quadratic (Q) orthogonal polynomial contrasts.

The LMA was not affected (*P* > 0.14) by the DDGS level in the diets, ratifying the finds of Schauer et al. (2008) and Felix et al. (2012). However, BFT (*P* = 0.04) and BWT (*P* < 0.001) quadratically increased. Yield grade (YG), estimated by BFT (USDA, 1992), was 2.2, 2.4, 2.6, and 1.7 for experimental groups 0DDGS, 25DDGS, 50DDGS, and 75DDGS, respectively. A greater YG reduces boneless retail cuts (USDA, 1992). However, the total body lean seems to be an appropriate means of differentiating dietary effects on carcass value (Felix et al., 2012). In this regard, boneless closely trimmed retail cuts (BCTRC), estimated using HCW, REA, and BWT data, were approximately 48 ± 0.35%, without any significant variation among the experimental diets.

The LC tended to quadratically increase (*P* = 0.06) with the DDGS levels in the diets. The leg is the most valuable part of the carcass, accounting for its weight and price (Notter et al., 2012). The LC affects leg score and carcass conformation, consequently the final grade and retail value (USDA, 1992).

Research on DDGS substituting traditional sources of protein and energy in feedlot lamb diets found no differences in major carcass traits (Van Emon et al., 2012; Abdelrahim et al., 2014; Kawęcka et al. 2018), which can be understood as an advantage to the sheep industry by reducing production costs without losing carcass grade (Schauer et al. 2008; Whitney and Braden, 2010; Crane et al., 2017). However, in agreement with this study, some authors (Felix et al. 2012; Curzaynz-Leyva et al. 2019) have reported positive effects of DDGS levels on lamb carcass traits, which might be even more interesting for the sheep industry.

### Adipose Tissue FA Characteristics

Differences in the fat percentage and most adipose tissue FA, including the saturated fatty acids (SFA) palmitic and lauric, were not detected when the DDGS level was increased in the experimental diets (Table 5). However, some major FA in the lamb meat had remarkable alterations. Increasing DDGS in the diets linearly (*P* = 0.02) increased stearic acid (C18:0) due to its dietary increase and principally because of the conversion of linoleic acid (C18:2), the predominant FA in DDGS, into C18:0, which increases C18:0 duodenal flow (Castillo-Lopez et al, 2014; Giotto et al., 2020). Rumen microorganisms hydrogenate a substantial proportion of PUFA (i.e., C18:2) contained in ruminant diets, resulting in high levels of SFA deposition in muscle, including stearic acid (Wood et al., 2004). In addition, increased NDF in the diets with DDGS may have increased the extension of C18:2 biohydrogenation (Sackman et al., 2003; Santos-Silva et al., 2018) because this process is related primarily to cellulolytic microorganisms (Kepler and Tove, 1967), and feeding DDGS may favor their population growth in the rumen (Depenbusch et al., 2009; Kawęcka et al., 2018).

Linoleic acid, which is a PUFA and part of the ω-6 family, quadratically (*P* = 0.01) increased with DDGS levels, agreeing with Kawęcka et al. (2018) and Karaca et al. (2021). The utilization of DDGS in ruminant diets usually leads to the modification of meat FA profile since some FA may be protected from biohydrogenation (Vander Pol et al., 2009; Whitney and Braden, 2010; Giotto et al., 2020). The protection of dietary PUFA from ruminal biohydrogenation is a strategy for improving the FA profile of meat and milk (Bessa et al., 2015; Salami et al., 2021). Feeding DDGS may increase dietary fat digestibility and the amount of unsaturated FA reaching the distal gut, indicative of decreased susceptibility of dietary PUFA to ruminal biohydrogenation (Xu et al., 2014; Giotto et al., 2020). However, final concentrations of linoleic acid and other PUFA in muscle also depend on fiber types and the expression of desaturases in the tissue (Wood et al., 2008), knowing that desaturases convert SFA into unsaturated FA in the muscle and may also be regulated by dietary lipid content (Waters et al., 2009).

Oleic acid, a monounsaturated fatty acid (MUFA) ω-9, tended to (*P* = 0.08) quadratically decrease as DDGS in the diets increased. Oleic acid, which is the most represented FA in lamb meat, was proportionally 25% greater in meat than in the diets, and its inclusion in the human diet can bring health benefits, such as the reduction in blood low-density lipoproteins without reducing high-density lipoproteins (Polidori et al., 2011).

Diet influenced SFA, with the lowest value in the 50DDGS experimental group (49.0%) and the highest value in the 75DDGS experimental group (52.8%). The opposite occurred for PUFA with 13.3% and 8.1% for the 50DDGS and 75DDGS experimental groups, respectively. Although the utilization of DDGS in lamb diets may reduce SFA and increase PUFA and MUFA (Yossifov, 2014), the heterogeneous composition of DDGS, notably regarding its fat content (Liu, 2011), is a factor that should be considered in result comparisons because it may affect the meat’s FA profile (Scollan et al., 2017). Greater PUFA/SFA and MUFA + PUFA/SFA ratios were observed in lambs fed in the 50DDGS treatment group (0.27 and 0.97, respectively) compared to the lambs fed in the 0DDGS, 25DDGS, and 75DDGS treatment groups (0.17 and 0.93; 0.19 and 0.87; 0.15 and 0.85, respectively). From the consumers’ perspective, greater PUFA/SFA and MUFA + PUFA/SFA ratio can be associated with multiple health benefits (Polidori et al., 2011; Oliveira et al., 2015; Siri-Tarino et al., 2015).

### The sensory panel evaluation

The sensorial panel test results, except for cook-loss (*P* > 0.49) and off-flavor (*P* > 0.31) which had no significant differences, indicated that incorporating DDGS in lamb feedlot diets resulted in more acceptable lamb meat (Table 6). Increasing the DDGS levels in the diets quadratically increased the initial and sustained juiciness (*P* < 0.02) and tenderness (*P* < 0.009). According to Hodges et al. (2020b), although the addition of DDGS to feedlot lamb diets did not affect muscle fiber tenderness and connective tissue amount, it linearly increased meat tenderness. The increase of carcass backfat thickness and body wall from lambs fed with 25DDGS and 50DDGS may have reduced cold shortening (Smith and Carpenter, 1973) and its effects on decreasing sarcomere length and increasing shear force (Aalhus et al., 2001; Okeudo and Moss, 2005), which can be perceived by sensory panelists (Destefanis et al., 2008).

**Table 6.** Effects of substituting dried distillers` grains with solubles (DDGS) for cottonseed meal and sorghum grain on sensory panel traits of lamb Longissimus muscle chops

Item^1^	Diet^2^				SEM^3^	*P*-value^4^	
	0DDGS	25DDGS	50DDGS	75DDGS		L	Q
Cook-loss, %	6.7	6.1	8.2	6.5	0.8	0.67	0.49
Initial juiciness	5.5	6.1	6.2	5.9	0.2	0.07	0.02
Sustained juiciness	5.3	6.3	6.1	5.7	0.2	0.28	0.001
Initial tenderness	5.2	6.4	6.0	5.9	0.2	0.06	0.003
Sustained tenderness	5.1	6.3	6.1	6.0	0.2	0.01	0.009
Flavor intensity	5.1	5.4	5.4	5.6	0.1	0.005	0.82
Off-flavor	3.8	3.9	3.9	3.9	0.1	0.42	0.31
Overall acceptability	5.0	6.2	5.9	5.8	0.2	0.006	0.001

Cook loss expressed as a percentage of weight loss from raw weight; juiciness, tenderness, and flavor intensity (1 = extremely dry, tough, and bland, and 8 = extremely juicy, tender, and intense, respectively; off-flavor (1 = extreme off-flavor and 4 = no off-flavor); overall acceptability (1 = not acceptable; 8 = highly acceptable).

Lambs were fed pelleted diets containing DDGS that replaced 0% (0DDGS), 25% (25DDGS), 50% (50DDGS), or 75% (75DDGS) of the cottonseed meal and sorghum grain.

SEM represents the greatest standard error of the mean.

Linear (L) and quadratic (Q) orthogonal polynomial contrasts.

Increasing the DDGS levels in the diets quadratically increased the overall acceptability (*P* < 0.001) of the lamb chops. In addition, the DDGS levels linearly increased flavor intensity (*P* = 0.005), probably influenced by the positive correlation with initial and sustained juiciness and tenderness scores (Whitney and Braden, 2010). Juiciness, tenderness, and flavor scores can be affected by the inability of panelists to completely separate these traits, known a halo effect (Roeber et al., 2000). Consequently, increased sustained juiciness creates a generalized notion of increased tenderness and flavor.

The 25DDGS treatment group had the best overall sensorial panel results, with few differences from the 50DDGS treatment group. In general, these results were in agreement with those obtained by Whitney and Braden (2010), who tested the substitution of DDGS for CSM in feedlot Rambouillet lamb diets.

Chemical reactions (i.e., lipid oxidation) during lamb cooking generate volatile compounds responsible for the lamb flavor (Resconi et al., 2010). The taste and/or aroma derived from these volatile compounds is affected by the relative proportions of meat FA (Khan et al., 2015; Gkarane et al. 2019). Although increased PUFA is interesting for human health (Siri-Tarino et al., 2015), they may result in the loss of meat sensory quality (Elmore et al., 2005). For this reason, it can be hypothesized that the chops from lambs fed in the 50DDGS treatment group, which were richer in unsaturated FA (Table 5) had a slightly inferior performance in the sensory panel than the lambs in the 25DDGS treatment group (Table 6). Most carbonyl, aldehyde, and alcohol volatile compounds originate from PUFA oxidation during cooking and can be powerful odorant compounds (Resconi et al., 2010).

The average cook-loss was approximately 6.9%, which was less than that observed by Smeti et al. (2014) in meat from fat-tail Babarine lambs finished in feedlots and rangelands (8%–11%). These authors found that lambs finished in a feedlot produced meat with a greater score of juiciness, which can be negatively correlated with cook loss. The DDGS inclusion in feedlot lamb diets benefited lamb meat sensory properties, particularly tenderness and taste desirability, and can therefore meet consumer expectations in many different markets (Kawęcka et al., 2018). Using intermediate levels (25%–50%) of DDGS to substitute CSM and SG in feedlot lamb diets will likely produce meat with both desirable characteristics in terms of the FA profile and the consumers’ palate satisfaction.

## CONCLUSIONS

Replacing CSM and SG with DDGS in feedlot lamb diets increased growth performance and kept the gain:feed ratio unaltered, which can be advantageous for the sheep industry. However, urolith development could be problematic if proper Ca:P ratios are not maintained. Wool production and most of the wool’s quality parameters were not affected by the DDGS level. Carcass and meat quality traits, including the FA profile and the sensorial panel, were improved with the inclusion of DDGS in the diets, with better overall results when intermediate levels of substitution (25%–50%) were adopted. Finally, considering both animal performance and the quality of the final products, it is suggested the substitution of 50% of CSM and SG with DDGS.

## Abbreviations

AA: amino acids
ADF: acid detergent fiber
ADG: average daily gain
BCTRC: boneless closely trimmed retail cuts
BFT: backfat thickness
BUN: blood urea nitrogen
BW: body weight
BWT: body wall thickness
Ca: calcium
CF: crude fat
CP: crude protein
CSM: cottonseed meal
DDGS: dried distillers’ grains with solubles
DM: dry matter
DMI: dry matter intake
FA: fatty acid
FAME: fatty acid methyl ester
G:F: gain:feed
HCW: hot carcass weight
IGF-1: blood insulin-like growth factor-1
LM: longissimus muscle
LMA: longissimus muscle area
MUFA: monounsaturated fatty acid
N: nitrogen
NDF: neutral detergent fiber
P: phosphorus
PUFA: polyunsaturated fatty acid
RDP: rumen degradable protein
RUP: rumen undegradable protein
S: sulfur
SBM: soybean meal
SFA: saturated fatty acids
SG: sorghum grain
TDN: total digestible nutrients

## References

[CIT0001] Aalhus, J. L., J. A. Janz, A. K. Tong, S. D. Jones, and W. M. Robertson. 2001. The influence of chilling rate and fat cover on beef quality. Can. J. Anim. Sci. 81:321–330. doi:10.4141/A00-084.

[CIT0002] Abdelrahim, G. M., J. Khatiwada, and N. K. Gurung. 2014. Effects of dried distillers with solubles on performance and carcass characteristics of lamb. J. Anim. Res. Technol. 1:25–30. doi:10.5147/jart.v1i2.106.

[CIT0003] Alshdaifat, S. N., and B. S. Obeidat. 2019. The impact of feeding corn dried distillers grains with solubles on milk yield and composition in lactating Awassi ewes and digestibility and N partitioning in Awassi ewe lambs. Italian J. Anim. Sci. 18:522–529. doi:10.1080/1828051X.2018.1547126.

[CIT0004] AMSA. 1995. Research guidelines for cookery, sensory evaluation, and instrumental tenderness measurements of meat. Chicago, IL: American Meat Science Assoc.

[CIT0005] AOAC. 2006. Official methods of analysis, 18th ed. Arlington, VA: Assoc. Offic. Anal. Chem.

[CIT0006] Archibeque, S. L., D. K. Lunt, C. D. Gilbert, R. K. Tume, and S. B. Smith. 2005. Fatty acid indices of stearoyl-CoA desaturase do not reflect actual stearoyl-CoA desaturase enzyme activities in adipose tissues of beef steers finished with corn-, flaxseed-, or sorghum-based diets. J. Anim. Sci. 83:1153–1166. doi:10.2527/2005.8351153x. 15827260

[CIT0007] ASTM. 2007. Annual book of American Society for Testing and Materials standards. West Conshohocken, PA: Am. Soc. Test. Mat.

[CIT0008] ASTM. 2008. Annual book of American Society for Testing and Materials standards. West Conshohocken, PA: Am. Soc. Test. Mat.

[CIT0009] Baxter, B. P., M. A. Brims, and T. B. Taylor. 1992. Description and performance of the optical fibre diameter analyser (OFDA). J. Text. Inst. 83:507–526.

[CIT0010] Belyea, R. L., K. D. Rausch, T. E. Clevenger, V. Singh, D. B. Johnston, and M. E. Tumbleson. 2010. Sources of variation in composition of DDGSS. Anim. Feed Sci. Technol. 159:122–130. doi:10.1016/j.anifeedsci.2010.06.005.

[CIT0011] Berrie, R. A., D. M. Hallford, and M. L. Galyean. 1995. Effects of zinc source and level on performance and metabolic hormone concentrations of growing and finishing lambs. Prof. Anim. Sci. 11:149–156. doi:10.15232/S1080-7446(15)32579-1.

[CIT0012] Bessa, R. J., S. P. Alves, and J. Santos-Silva. 2015. Constraints and potentials for the nutritional modulation of the fatty acid composition of ruminant meat. Eur. J. Lipid Sci. Technol. 117:1325–1344. doi:10.1002/ejlt.201400468.

[CIT0013] Böttger, C., and K. H. Südekum. 2018. Review: protein value of distillers dried grains with solubles (DDGSS) in animal nutrition as affected by the ethanol production process. Anim. Feed Sci. Technol. 244:11–17. doi:10.1016/j.anifeedsci.2018.07.018.

[CIT0014] Bravo, D., D. Sauvant, C. Bogaert, and F. Meschy. 2003. III. Quantitative aspects of phosphorus excretion in ruminants. Reprod. Nutr. Dev. 43:285–300. doi:10.1051/rnd:2003021. 14620634

[CIT0015] Castillo-Lopez, E., H. A. Ramirez-Ramirez, T. J. Klopfenstein, C. L. Anderson, N. D. Aluthge, S. C. Fernando, T. Jenkins, and P. J. Kononoff. 2014. Effect of feeding dried distillers grains with solubles on ruminal biohydrogenation, intestinal fatty acid profile, and gut microbial diversity evaluated through DNA pyro-sequencing. J. Anim. Sci. 92:733–743. doi:10.2527/jas.2013-7223. 24664563

[CIT0016] Castro-Pérez, B. I., A. Estrada-Angulo, F. G. Ríos, H. Dávila-Ramos, J. C. Robles-Estrada, G. Contreras-Pérez, J. F. Calderón-Cortés, M. A. López-Soto, A. Barreras, and A. Plascencia. 2014. Effects of replacing partially dry-rolled corn and soybean meal with different levels of dried distillers grains with solubles on growth performance, dietary energetics, and carcass characteristics in hairy lambs fed a finishing diet. Small Rumin. Res. 119:8–15. doi:10.1016/j.smallrumres.2014.03.007.

[CIT0017] Crane, A. R., R. R. Redden, K. C. Swanson, B. M. Howard, T. J. Frick, K. R. Maddock-Carlin, and C. S. Schauer. 2017. Effects of dried distiller’s grains and lasalocid inclusion on feedlot lamb growth, carcass traits, nutrient digestibility, ruminal fluid volatile fatty acid concentrations, and ruminal hydrogen sulfide concentration. J. Anim. Sci. 95:3198–3205. doi:10.2527/jas.2017.1369. 28727092

[CIT0018] Cross, H. R., R. Moen, and M. S. Stanfield. 1978. Training and testing of judges for sensory analysis of meat quality. Food Technol. 37:48–54.

[CIT0019] Curzaynz-Leyva, K. R., J. R. Bárcena-Gama, C. Sánchez-del Real, J. C. Escobar-España, M. I. Rivas-Martínez, E. A. Santillán-Gómez, D. F. Portela-Díaz, and E. J. Flores-Santiago. 2019. Effect of dried distillers grains (DDGS) on diet digestibility, growth performance, and carcass characteristics in Creole wool lambs fed finishing diets. S. Afr. J. Anim. Sci. 49:56–63. doi:10.4314/sajas.v49i1.7.

[CIT0020] Depenbusch, B. E., C. M. Coleman, J. J. Higgins, and J. S. Drouillard. 2009. Effects of increasing levels of dried corn distillers grains with solubles on growth performance, carcass characteristics, and meat quality of yearling heifers. J. Anim. Sci. 87:2653–2663. doi:10.2527/jas.2008-1496. 19359505

[CIT0021] Destefanis, G., A. Brugiapaglia, M. T. Barge, and E. Dal Molin. 2008. Relationship between beef consumer tenderness perception and Warner-Bratzler shear force. Meat Sci. 78:153–156. doi:10.1016/j.meatsci.2007.05.031. 22062265

[CIT0022] Elmore, J. S., S. L. Cooper, M. Enser, D. S. Mottram, L. A. Sinclair, R. G. Wilkinson, and J. D. Wood. 2005. Dietary manipulation of fatty acid composition in lamb meat and its effect on the volatile aroma compounds of grilled lamb. Meat Sci. 69:233–242. doi:10.1016/j.meatsci.2004.07.002. 22062813

[CIT0023] Felix, T. L., H. N. Zerby, S. J. Moeller, and S. C. Loerch. 2012. Effects of increasing dried distillers grains with solubles on performance, carcass characteristics, and digestibility of feedlot lambs. J. Anim. Sci. 90:1356–1363. doi:10.2527/jas.2011-4373. 22147466

[CIT0024] Folch, J., M. Lees, and G. H. Sloane-Stanley. 1957. A simple method for the isolation and purification of total lipids from animal tissues. J. Biol. Chem. 226:497–507. doi:10.1016/S0021-9258(18)64849-5. 13428781

[CIT0025] Giotto, F. M., A. P. Fruet, J. L. Nörnberg, C. R. Calkins, and A. S. Mello. 2020. Effects of muscle and finishing diets containing distillers grains with low moisture levels on fatty acid deposition in two novel value-added beef cuts. Food Sci. Anim. Resour. 40:484–494. doi:10.5851/kosfa.2020.e28. 32426725 PMC7207090

[CIT0026] Gkarane, V., N. P. Brunton, P. Allen, R. S. Gravador, N. A. Claffey, M. G. Diskin, A. G. Fahey, L. J. Farmer, A. P. Moloney, M. J. Alcalde, et al. 2019. Effect of finishing diet and duration on the sensory quality and volatile profile of lamb meat. Food Res. Int. 115:54–64. doi:10.1016/j.foodres.2018.07.063. 30599976

[CIT0027] Hassan, S. A., R. A. M. Al Jassim, A. N. Al-Ani, and N. S. Abdullah. 1991. Effects of dietary supplement of rumen un degradable protein upon carcass composition of fat-tail Awassi sheep. Small Rumin. Res. 5:65–74. doi: 10.1016/0921-4488(91)90031-K.

[CIT0028] Hodges, K. M., T. R. Whitney, W. S. Ramsey, and C. R. Kerth. 2020a. Replacing cottonseed meal and sorghum grain with corn dried distillers grains with solubles in lamb feedlot diets: growth performance, rumen fluid parameters, and blood serum chemistry. Sheep Goat Res. J. 35:1–9.

[CIT0029] Hodges, K. M., C. R. Kerth, T. R. Whitney, K. R. Wall, R. K. Miller, W. S. Ramsey, and D. R. Woerner. 2020b. Replacing cottonseed meal and sorghum grain with corn dried distillers’ grains with solubles in lamb feedlot diets: carcass, trained sensory painel, and volatile aroma compounds traits. J. Anim. Sci. 98:1–8. doi:10.1093/jas/skaa182. PMC7295328 32504491

[CIT0030] Hoffman, L.A., and A. Baker. 2011. Estimating the substitution of distillers` grains for corn and soybean meal in the U.S. feed complex. USDA report FDS11-I-01. Washington, DC: ERS, USDA.

[CIT0031] Huls, T. J., A. J. Bartosh, J. A. Daniel, R. D. Zelinsky, J. Held, and A. E. Wertz-Lutz. 2006. Efficacy of dried distiller’s grains with solubles as a replacement for soybean meal and a portion of the corn in a finishing lamb diet. Sheep Goat Res. J. 21:30–34.

[CIT0032] Huntington, G. B., and S. L. Archibeque. 2000. Practical aspects of urea and ammonia metabolism in ruminants. J. Anim. Sci. 77:1–11. doi:10.2527/jas2000.77E-Suppl1y.

[CIT0033] Jianu, S., V. Crivineanu, G. V. Goran, and L. Tudoreanu. 2013. Study of main blood minerals concentrations in different physiological states in cattle and sheep. Lucrări Ştiinţifice. 46:87–93.

[CIT0034] Johnson, C. L., and S. A. Larsen. 1978. Clean wool determination of individual fleeces. J. Anim. Sci. 47:41–45. doi:10.2527/jas1978.47141x.

[CIT0035] Karaca, S., S. Erdoğan, M. Güney, C. Çakmakçı, M. Sarıbey, A. Kor, and H. Ülker. 2021. Does the length of time dried distillers’ grain with solubles substitution for soybean meal affect physiological indicators and meat quality in finishing lambs? Anim. Sci. J. 92:e13561. doi:10.1111/asj.13561. 34018642

[CIT0036] Kawęcka, A., E. Sosin-Bzducha, M. Puchała, and J. Sikora. 2018. Effect of maize DDGSS addition on carcass and meat quality of lambs of native sheep breed. J. Appl. Anim. Res. 46:301–305. doi:10.1080/09712119.2017.1299014.

[CIT0037] Kepler, C. R., and S. B. Tove. 1967. Biohydrogenation of unsaturated fatty acids. 3. Purification and properties of a linoleate delta-12-cis, delta-11-trans-isomerase from Butyrivibrio fibrisolvens. J. Biol. Chem. 242:5686–5692. 5633396

[CIT0038] Kerth, C. R., L. K. Blair-Kerth, and W. R. Jones. 2003. Warner-Bratzler shear force repeatability in beef longissimus steaks cooked with a convection oven, broiler, or clamshell grill. J. Food Sci. 68:668–670. doi:10.1111/j.1365-2621.2003.tb05729.x.

[CIT0039] Khan, M. J., A. Abbas, M. Ayaz, M. Naeem, M. S. Akhter, and M. H. Soomro. 2012. Factors affecting wool quality and quantity in sheep. Afr. J. Biotechnol. 11:13761–13766. doi:10.5897/AJBX11.064.

[CIT0040] Khan, M. I., C. Jo, and M. R. Tariq. 2015. Meat flavor precursors and factors influencing flavor precursors: a systematic review. Meat Sci. 110:278–284. doi:10.1016/j.meatsci.2015.08.002. 26319308

[CIT0041] Kleinschmit, D. H., J. L. Anderson, D. J. Schingoethe, K. F. Kalscheur, and A. R. Hippen. 2007. Ruminal and intestinal degradability of distillers grains plus solubles varies by source. J. Dairy Sci. 90:2909–2918. doi:10.3168/jds.2006-613. 17517731

[CIT0042] Klopfenstein, T. 1996. Distillers grains as an energy source and effect of drying on protein availability. Anim. Feed Sci. Technol. 60:201–207. doi:10.1016/0377-8401(96)00978-9.

[CIT0043] Kohn, R. A., M. M. Dinneen, and E. Russek-Cohen. 2005. Using blood urea nitrogen to predict nitrogen excretion and efficiency of nitrogen utilization in cattle, sheep, goats, horses, pigs, and rats. J. Anim. Sci. 83:879–889. doi:10.2527/2005.834879x. 15753344

[CIT0044] Lima, N. L. L., C. R. F. Ribeiro, H. C. M. Sá, I. Leopoldino-Júnior, L. F. L. Cavalcanti, R. A. V. Santana, I. F. Furusho-Garcia, and I. G. Pereira. 2017. Economic analysis, performance, and feed efficiency in feedlot lambs. Braz. J. Anim. Sci. 46:821–829. doi:10.1590/S1806-92902017001000005.

[CIT0045] Liu, K. 2011. Chemical composition of distillers grains, a review. J. Agric. Food Chem. 59:1508–1526. doi:10.1021/jf103512z. 21299215

[CIT0046] Louvandini, H., and D. M. S. S. Vitti. 1996. Phosphorus metabolism and estimation of phosphorus requirements for sheep. Sci. Agric. 53:184–189. doi:10.1590/S0103-90161996000100027.

[CIT0047] Malau-Aduli, A. E. O., D. V. Nguyen, H. V. Le, Q. V. Nguyen, J. R. Otto, B. S. Malau-Aduli, and P. D. Nichols. 2019. Correlations between growth and wool quality traits of genetically divergent Australian lambs in response to canola or flaxseed oil supplementation. PLoS One 14:e0208229. doi:10.1371/journal.pone.0208229. 30605467 PMC6317778

[CIT0048] McEachern, J. K., T. R. Whitney, C. B. Scott, C. J. Lupton, and M. W. Salisbury. 2009. Substituting distillers dried grains for cottonseed meal in lamb-finishing diets: growth, wool characteristics, and serum NEFA, urea N, and IGF-1 concentrations. Sheep Goat Res. J. 24:32–40.

[CIT0049] Mikolayunas-Sandrock, C., L. E. Armentano, D. L. Thomas, and Y. M. Berger. 2009. Effect of protein degradability on milk production of dairy ewes. J. Dairy Sci. 92:4507–4513. doi:10.3168/jds.2008-1983. 19700712

[CIT0050] Mjoun, K., K. F. Kalscheur, A. R. Hippen, and D. J. Schingoethe. 2010. Ruminal degradability and intestinal digestibility of protein and amino acids in soybean and corn distillers grains products. J. Dairy Sci. 93:4144–4154. doi:10.3168/jds.2009-2883. 20723689

[CIT0051] Moreau, R. A., K. Liu, J. K. Winkler-Moser, and V. Singh. 2011. Changes in lipid composition during dry grind ethanol processing of corn. J. Am. Oil Chem. Soc. 88:435–442. doi:10.1007/s11746-010-1674-y.

[CIT0052] Morrison, W. R., and L. M. Smith. 1964. Preparation of fatty acid methyl esters and dimethylacetals from lipids with boron fluoride–methanol. J. Lipid Res. 5:600–607. doi:10.1016/S0022-2275(20)40190-7. 14221106

[CIT0053] Naderi, N., Z. Salehian, M. Souri, F. Hodjabri, and R. Mirmahmoudi. 2015. Influence of nutrition supplementation on the seasonal change in fiber growth and skin follicle activity in both male and female Sanjabi lambs. Small Rum. Res. 123:103–109. doi:10.1016/j.smallrumres.2014.10.004.

[CIT0054] NAMP. 1997. The meat buyers guide. Reston, VA: North Am. Meat Proc. Assoc.

[CIT0055] Nolan, E., T. Farrell, M. Ryan, C. Gibbon, and F. Z. Ahmadi-Esfahani. 2014. Valuing quality attributes of Australian merino wool. Aust. J. Agric. Resour. Econ. 58:314–335. doi:10.1111/1467-8489.12027.

[CIT0056] Notter, D. R., T. D. Leeds, M. R. Mousel, J. B. Taylor, D. P. Kirschten, and G. S. Lewis. 2012. Evaluation of Columbia, USMARC-Composite, Suffolk, and Texel rams as terminal sires in an extensive rangeland production system: II. Postweaning growth and ultrasonic measures of composition for lambs fed a high-energy feedlot diet. J. Anim. Sci. 90:2941–2952. doi:10.2527/jas.2011-4641. 22408090

[CIT0057] NRC. 2007. Nutrient requirements of small ruminants: sheep, goats, cervids, and new world camelids. Washington, DC: National Academic Press.

[CIT0058] Nuez Ortín, W. G., and P. Yu. 2009. Nutrient variation and availability of wheat DDGSS, corn DDGSS and blend DDGSS from bioethanol plants. J. Sci. Food Agric. 89:1754–1761. doi:10.1002/jsfa.3652.

[CIT0059] Obeidat, B. S. 2018. Influence of corn-dried distiller’s grain with solubles on growth performance and blood metabolites of Awassi lambs offered a concentrate diet. Italian J. Anim. Sci. 17:636–642. doi:10.1080/1828051X.2017.1404946.

[CIT0060] Okeudo, N. J., and B. W. Moss. 2005. Interrelationships amongst carcass and meat quality characteristics of sheep. Meat Sci. 69:1–8. doi:10.1016/j.meatsci.2004.04.011. 22062633

[CIT0061] Oliveira, V., R. Marinho, D. Vitorino, G. A. Santos, J. C. Moraes, N. Dragano, A. Sartori-Cintra, L. Pereira, R. R. Catharino, A. S. R. Silva, et al. 2015. Diets containing α-linolenic (ω3) or oleic (ω9) fatty acids rescues obese mice from insulin resistance. Endocrinology. 156:4033–4046. doi:10.1210/en.2014-1880. 26280128

[CIT0062] Peripolli, V., E. R. Prates, J. O. J. Barcellos, and J. Braccini Neto. 2011. Fecal nitrogen to estimate intake and digestibility in grazing ruminants. Anim. Feed Sci. Technol. 163:170–176. doi:10.1016/j.anifeedsci.2010.11.008.

[CIT0063] Piccione, G., A. Foa, C. Bertolucci, and G. Caola. 2006. Daily rhythm of salivary and serum concentration of sheep. J. Circadian Rhythm. 4:16–19. doi:10.1186/1740-3391-4-16. PMC1664584 17123442

[CIT0064] Polidori, P., A. Ortenzi, S. Vincenzetti, and D. Beghelli. 2011. Dietary properties of lamb meat and human health. Mediterr. J. Nutr. Metab. 4:53–56. doi:10.1007/s12349-010-0032-9.

[CIT0065] Quadros, D. G., T. R. Whitney, D. R. Tolleson, and C. R. Kerth. 2021. Incorporating dried distillers grains with solubles in sheep supplementation programs. Sheep Goat Res. J. 36:8–25.

[CIT0066] Radev, V. 2012. Effect of dietary supplementation of dried distillers grains with solubles (Zarnela) on some rumen fermentation parameters in yearling sheep. Agric. Sci. Technol. 4:241–245.

[CIT0067] Resconi, V. C., M. M. Campo, F. Montossi, V. Ferreira, C. Sañudo, and A. Escudero. 2010. Relationship between odour-active compounds and flavour perception in meat from lambs fed different diets. Meat Sci. 85:700–706. doi:10.1016/j.meatsci.2010.03.027. 20416794

[CIT0068] Riedi, A. K., G. Knubben-Schweizer, and M. Meylan. 2018. Clinical findings and diagnostic procedures in 270 small ruminants with obstructive urolithiasis. J. Vet. Intern. Med. 32:1274–1282. doi:10.1111/jvim.15128. 29660779 PMC5980268

[CIT0069] Roeber, D. L., R. C. Cannell, K. E. Belk, R. K. Miller, J. D. Tatum, and G. C. Smith. 2000. Implant strategies during feeding: impact on carcass grades and consumer acceptability. J. Anim. Sci. 78:1867–1874. doi:10.2527/2000.7871867x. 10907829

[CIT0070] Ružić-Muslić, D., M. P. Petrovic, M. M. Petrovic, Z. Bijelic, V. Pantelic, and P. Perišic. 2016. Effects of different protein sources of diet on yield and quality of lamb meat. Afr. J. Biotechnol. 10:15823–15829. doi:10.5897/AJB11.698.

[CIT0071] Şahin, T., O. Kaya, D. Aksu Elmali, and I. Kaya. 2013. Effects of dietary supplementation with distiller dried grain with solubles in growing lambs on growth, nutrient digestibility and rumen parameters. Rev. Med. Vet. (Toulouse). 164:173–178.

[CIT0072] Sackman, J. R., S. K. Duckett, M. H. Gillis, C. E. Realini, A. H. Parks, and R. B. Eggelston. 2003. Effects of forage and sunflower oil levels on ruminal biohydrogenation of fatty acids and conjugated linoleic acid formation in beef steers fed finishing diets. J. Anim. Sci. 81:3174–3181. doi:10.2527/2003.81123174x. 14677873

[CIT0073] Salami, S. A., M. N. O’Grady, G. Luciano, A. Priolo, M. McGee, A. P. Moloney, and J. P. Kerry. 2021. Fatty acid composition, shelf-life and eating quality of beef from steers fed corn or wheat dried distillers’ grains with solubles in a concentrate supplement to grass silage. Meat Sci. 173:108381. doi:10.1016/j.meatsci.2020.108381. 33288361

[CIT0074] Santos-Silva, J., A. Francisco, S. P. Alves, P. Portugal, T. Dentinho, J. Almeida, D. Soldado, E. Jerónimo, and R. J. B. Bessa. 2018. Effect of dietary neutral detergent fibre source on lambs growth, meat quality and biohydrogenation intermediates. Meat Sci. 147:28–36. doi:10.1016/j.meatsci.2018.08.015. 30196198

[CIT0075] Schauer, C. S., M. M. Stamm, T. D. Maddock, and P. B. Berg. 2008. Feeding of DDGSS in lamb rations: feeding dried distillers grains with solubles as 60 percent of lamb finishing rations results in acceptable performance and carcass quality. Sheep Goat Res. J. 23:15–19.

[CIT0076] Scollan, N. D., E. M. Price, S. A. Morgan, S. A. Huws, and K. J. Shingfield. 2017. Can we improve the nutritional quality of meat? Proc. Nutr. Soc. 76:603–618. doi:10.1017/S0029665117001112. 28942754

[CIT0077] Siri-Tarino, P. W., S. Chiu, N. Bergeron, and R. M. Krauss. 2015. Saturated fats versus polyunsaturated fats versus carbohydrates for cardiovascular disease prevention and treatment. Annu. Rev. Nutr. 35:517–543. doi:10.1146/annurev-nutr-071714-034449. 26185980 PMC4744652

[CIT0078] Smeti, S., M. Mahouachi, and N. Atti. 2014. Effects of finishing lambs in rich aromatic plant pasture or in feedlot on growth and meat quality. J. Appl. Anim. Res. 42:297–303. doi:10.1080/09712119.2013.845102.

[CIT0079] Smith, G. C., and Z. L. Carpenter. 1973. Postmortem shrinkage of lamb carcasses. J. Anim. Sci. 36:862–867. doi:10.2527/jas1973.365862x.

[CIT0080] Song, S., J. Wu, S. Zhao, D. P. Casper, L. Zhang, B. He, X. Lang, C. Wang, X. Gong, F. Wand, et al. 2018. The effect of periodic energy restriction on growth performance, serum biochemical indices, and meat quality in sheep. J. Anim. Sci. 96:4251–4263. doi:10.1093/jas/sky299. 30247690 PMC6162581

[CIT0081] Spiehs, M. J., M. H. Whitney, and G. C. Shurson. 2002. Nutrient database for distiller’s dried grains with solubles produced from new ethanol plants in Minnesota and South Dakota. J. Anim. Sci. 80:2639–2645. doi:10.2527/2002.80102639x. 12413086

[CIT0082] Stojković, J., Z. Ilić, M. Milenković, R. Đoković, and B. Jašović. 2011. The impact of physiological condition on calcium, phosphorus and magnesium levels in sheep blood serum. Biotechnol. Anim. Husb. 27:1113–1121. doi:10.2298/BAH1404601S.

[CIT0083] Swanson, K. C., J. S. Caton, D. A. Redner, V. I. Burke, and L. P. Reynolds. 2000. Influence of undegraded intake protein on intake, digestion, serum hormones and metabolites, and nitrogen balance in sheep. Small Rum. Res. 35:225–233. doi:10.1016/S0921-4488(99)00091-7.

[CIT0084] Trujillo, A. T., M. Bruni, and P. Chilibroste. 2016. Nutrient content and nutrient availability of sorghum wet distiller’s grain in comparison with the parental grain for ruminants. J. Sci. Food Agric. 97:2353–2357. doi:10.1002/jsfa.8046. 27653319

[CIT0085] USDA. 1992. United States standards for grades of lamb, yearling mutton, and mutton carcasses. Washington, DC: AMS, USDA.

[CIT0086] USDA. 1997. Official United States Standards for grades of carcass beef. Washington, DC: AMS, USDA.

[CIT0087] USDA. 2021. U.S. Bioenergy statistics: co-products, dried distillers grain with solubles supply and disappearance. Washington, DC: ERS, USDA.

[CIT0088] Vander Pol, K. J., M. K. Luebbe, G. I. Crawford, G. E. Erickson, and T. J. Klopfenstein. 2009. Performance and digestibility characteristics of finishing diets containing distillers grains, composites of corn processing coproducts, or supplemental corn oil. J. Anim. Sci. 87:639–652. doi:10.2527/jas.2008-1036. 18952733

[CIT0089] Van Emon, M. L., P. J. Gunn, M. K. Neary, R. P. Lemenage, A. F. Schultz, and S. L. Lake. 2012. Effects of added protein and dietary fat on lamb performance and carcass characteristics when fed differing levels of dried distiller’s grains with soluble. Small Rum. Res. 103:164–168. doi:10.1016/j.smallrumres.2011.09.002.

[CIT0090] Van Soest, P. J., J. B. Robertson, and B. A. Lewis. 1991. Methods for dietary fiber, neutral detergent fiber, and nonstarch polysaccharides in relation to animal nutrition. J. Dairy Sci. 74:3583–3597. doi:10.3168/jds.S0022-0302(91)78551-2. 1660498

[CIT0091] Villalba, J. J., F. D. Provenza, and J. O. Hall. 2008. Learned appetites for calcium, phosphorus and sodium in sheep. J. Anim. Sci. 86:738–747. doi:10.2527/jas.2007-0189. 18073279

[CIT0092] Waters, S. M., J. P. Kelly, P. O’Boyle, A. P. Moloney, and D. A. Kenny. 2009. Effect of level and duration of dietary n-3 polyunsaturated fatty acid supplementation on the transcriptional regulation of Δ9-desaturase in muscle of beef cattle. J. Anim. Sci. 97:244–252. doi:10.2527/jas.2008-1005. 18791145

[CIT0093] Whitney, T. R., and K. W. Braden. 2010. Substituting corn dried distillers grains for cottonseed meal in lamb finishing diets: carcass characteristics, meat fatty acid profiles, and sensory panel traits. Sheep Goat Res. J. 25:49–56.

[CIT0094] Whitney, T. R., and C. J. Lupton. 2010. Evaluating percentage of roughage in lamb finishing diets containing 40% dried distillers grains: growth, serum urea nitrogen, nonesterified fatty acids, and insulin growth factor-1 concentrations and wool, carcass, and fatty acid characteristics. J. Anim. Sci. 88:3030–3040. doi:10.2527/jas.2010-2875. 20495127

[CIT0095] Whitney, T. R., and J. Muir. 2010. Redberry juniper as a roughage source in lamb feedlot rations: performance and serum nonesterified fatty acids, urea nitrogen, and insulin-like growth factor-1 concentrations. J. Anim. Sci. 88:1492–1502. doi:10.2527/jas.2009-2410. 19966150

[CIT0096] Whitney, T. R., C. J. Lupton, J. P. Muir, R. P. Adams, and W. C. Stewart. 2014. Effects of using ground redberry juniper and dried distillers grains with solubles in lamb feedlot diets: growth, blood serum, fecal, and wool characteristics. J. Anim. Sci. 92:1119–1132. doi:10.2527/jas.2013-7007. 24492543

[CIT0097] Wolf, A., M. Watson, and N. Wolf. 2003. Digestion and dissolution methods for P, K, Ca, Mg, and trace elements. In: J. Peters, editor, Recommended methods for manure analysis. Madison, WI: University Wisconsin Extension. p. 30–38.

[CIT0098] Wood, J. D., R. I. Richardson, G. R. Nute, A. V. Fisher, M. M. Campo, E. Kasapidou, P. R. Sheard, and M. Enser. 2004. Effects of fatty acids on meat quality: a review. Meat Sci. 66:21–32. doi:10.1016/S0309-1740(03)00022-6. 22063928

[CIT0099] Wood, J. D., M. Enser, A. V. Fisher, G. R. Nute, P. R. Sheard, R. I. Richardson, S. I. Hughes, and F. M. Whittington. 2008. Fat deposition, fatty acid composition and meat quality: a review. Meat Sci. 78:343–358. doi:10.1016/j.meatsci.2007.07.019. 22062452

[CIT0100] Xu, L., Y. Jin, M. He, C. Li, K. Beauchemin, and W. Yang. 2014. Effects of increasing levels of corn dried distillers grains with solubles and monensin on ruminal biohydrogenation and duodenal flows of fatty acids in beef heifers fed high-grain diets. J. Anim. Sci. 92:1089–1098. doi:10.2527/jas.2013-6668. 24492547

[CIT0101] Yang, C., J. Zhang, A. A. Ahmad, P. Bao, X. Guo, R. Long, X. Ding, and P. Yan. 2019. Dietary energy levels affect growth performance through growth hormone and insulin-like growth factor 1 in Yak (*Bos grunniens*). Animals (Basel). 9:1–13. doi:10.3390/ani9020039. PMC6406270 30696034

[CIT0102] Yossifov, M. R. 2014. Dietary influence on fatty acid characteristics of lamb carcass in relation to protein source. Biotechnol. Anim. Husb. 30:203–214.

