# Correction to: Replacing cottonseed meal and sorghum with dried distillers’ grains with solubles enhances the growth performance, carcass traits, and meat quality of feedlot lambs

**DOI:** 10.1093/tas/txad025

**Published:** 2023-03-30

Translational Animal Science, 2022 (https://doi.org/10.1093/tas/txac040)

The author list has been amended to Danilo G Quadros and Chris R Kerth, because another individual originally ­included did not consent to being named as a co-author of this article.

